# Deciphering the groove-binding mode of dolutegravir with salmon sperm DNA through spectroscopic and molecular modelling approaches

**DOI:** 10.1038/s41598-026-40136-y

**Published:** 2026-03-13

**Authors:** Eman Yosrey, Mohammad A. Elmorsy, Heba Elmansi, Shereen Shalan, Jenny Jeehan Nasr

**Affiliations:** 1 Department of Pharmaceutical Analytical Chemistry, Faculty of Pharmacy, Mansoura University, Mansoura, 35516 Egypt; 2 Department of Pharmaceutical Organic Chemistry, Faculty of Pharmacy, Mansoura University, Mansoura, 35516 Egypt; 3 Department of Pharmaceutical Chemistry, Faculty of Pharmacy, Delta University for Science and Technology, Gamasa City, 11152 Dakahliya Egypt

**Keywords:** Salmon sperm DNA, Dolutegravir, Binding interaction, Spectroscopy, Docking, Biochemistry, Biophysics, Chemical biology, Chemistry, Computational biology and bioinformatics, Drug discovery

## Abstract

Understanding how small molecules interact with DNA opens new avenues for designing smarter, more selective therapies. These studies not only shed light on off-target effects that could cause side effects or influence treatment outcomes but also help predict a drug’s genotoxic potential, aiding long-term safety assessments. Dolutegravir (DGV) serves as a second-generation integrase inhibitor used as a first-line antiretroviral therapy for managing human immunodeficiency virus (HIV) infection. Recent studies have positioned DGV as a prospective lead compound for repositioning antiretrovirals as cancer treatments. Building on this perspective, the introduced protocol presents a detailed approach to exploring DGV’s genomic interactions using salmon sperm DNA (SS-DNA) as a reliable genomic surrogate, employing various biophysical methods, including spectroscopic analysis, viscosity profiling, ionic strength experiments, and molecular docking. UV-Visible results indicate that DGV binds to DNA grooves with a binding constant of 10^3^ M⁻¹, as determined by the modified Benesi–Hildebrand equation. Fluorescent displacement assays with ethidium bromide and rhodamine B confirm the groove interaction mode with SS-DNA. Potassium iodide quenching of DGV yielded comparable quenching constants of 24.99 and 23.61 M⁻¹ in the presence and absence of DNA, respectively, giving a confirmatory sign for groove binding interaction. A constant viscosity profile after the addition of DGV provides strong evidence of the groove binding mechanism, while ionic strength assays ruled out any significant electrostatic contribution. In silico molecular docking further shows DGV’s preference for GC-rich regions of SS-DNA. Thermodynamic measurements taken at various temperatures indicate that the interaction is spontaneous (∆G° = -15.0 to -25.4 kJ mol^− 1^) and primarily driven by hydrogen bonds and van der Waals forces (∆H° = -198.51 kJ mol^− 1^ and ∆S° = -573.33 J mol^− 1^ K^− 1^). Overall, this work provides a foundational framework and a pioneering step for future clinical and pharmacological research, as well as genome integrity assessments, with the ultimate goal of developing DNA-targeted drugs with higher selectivity and effectiveness.

**Supplementary Information:**

The online version contains supplementary material available at 10.1038/s41598-026-40136-y.

## Introduction

Dolutegravir (DGV, Fig. S1) is described chemically as (3S,7R)-N-[(2,4-difluorophenyl)methyl]−11-hydroxy-7-methyl-9,12-dioxo-4-oxa-1,8-diazatricyclo [8.4.0.03,8] tetradeca-10,13-diene-13-carboxamide^1^. DGV is classified as a second-generation integrase strand transfer inhibitor and represents a cornerstone of first-line antiretroviral therapy for the treatment of human immunodeficiency virus (HIV) infection^2^. It exerts its antiviral activity by binding to the active site of HIV integrase, thereby inhibiting the strand transfer step required for the integration of viral DNA into the host genome. Since this step is critical in the HIV replication cycle, its inhibition effectively suppresses viral replication and reduces viral activity^2^. In clinical practice, DGV is available as a 50 mg single-agent formulation or as a fixed-dose combination with tenofovir and either lamivudine or emtricitabine.

Recent studies have positioned DGV as a prospective candidate for repurposing antiretrovirals as cancer treatments. Clinical investigations have demonstrated that DGV effectively suppresses the growth of various cancer cell lines, particularly those of prostate origin. Its antiproliferative activity is closely linked to inhibiting the expression of human endogenous retrovirus type K (HERV-K) in cancer cells^3^. These findings highlight the significance of investigating its interaction with DNA. Despite its clinical relevance, the interaction of DGV with host DNA remains unexplored, raising questions about potential off-target effects and long-term safety. Although DGV mainly targets viral integrase rather than host DNA directly, investigating its possible interactions with nucleic acids remains essential. This study not only deepens insight into DGV’s pharmacodynamics but also helps check secondary mechanisms that might impact its efficacy. Moreover, the study provides vital information about its genotoxicity profile and helps predict its safety for long-term use.

Deoxyribonucleic acid (DNA) is a fundamental macromolecule that serves as the repository of genetic information that orchestrates a wide range of essential physiological functions. It encodes hereditary data and regulates the production of proteins and enzymes involved in transport, replication, and the interpretation of genetic information. Alongside its central functions in genetic transmission and protein synthesis, DNA serves as a pivotal target in drug discovery and development, including antifungal, antiviral, and anticancer agents^4–6^.

Structurally, DNA consists of two biopolymer strands coiled into a double helix, forming the minor groove where strands are close together, and the major groove where they are more widely spaced. Drug-DNA interaction occurred through covalent and non-covalent mechanisms. Non-covalent interactions encompass intercalative, as well as non-intercalative modes such as electrostatic interactions and groove binding. In the intercalation binding mode, the drug molecules insert themselves between adjacent DNA base pairs, inducing conformational distortion of the DNA backbone. In contrast, the groove binding mode involves the insertion of drug molecules into the major or minor grooves of the DNA helix, exerting minimal to no impact on the backbone structure. Electrostatic interaction, on the other hand, occurs through the attraction between positively charged drug groups and the negatively charged DNA backbone^7–9^. Studying these interaction modes is a crucial area of research for elucidating the structural properties of DNA, understanding the pharmacology of certain drugs, and uncovering the molecular basis of certain diseases. Such studies not only reveal new structural motifs and identify promising molecular probes for chemical biology but also guide the rational design of new DNA-targeted therapeutics with improved efficacy and fewer side effects^10^.

Various DNA models have been used in DNA-binding studies, including salmon sperm DNA^8,9^, calf thymus DNA^11–14^, and herring sperm DNA^15^. These models are similar in structure, with 587–831 base pairs and comparable G-C and A-T content (41.9 mol% G-C and 58.1 mol% A-T). Consequently, their properties are quite alike. Owing to its virtues of being readily available and affordable, the salmon sperm DNA (SS-DNA) is commonly used in scientific research. Accordingly, it was selected as a reliable genomic surrogate to unravel the interaction profile with DGV.

Literature surveys revealed that the interaction of various small molecules with DNA has been widely investigated. Phyto-components, including isoeugenol^7^, eugenol^8^, capsaicin^16^, caffeic acid^17^, arbutin^18^, resveratrol, genistein, and curcumin^19^have been studied. Additionally, the interaction mechanisms of medications with different pharmacological actions, including ketoconazole^4^, posaconazole^10^, remdesivir^5^, palbociclib^6^, metformin^20^, and nintedanib^21^ have also been addressed.

Recent reports continue to emphasize the value of combining UV–Vis and fluorescence binding assays with computational modelling to rationalize small-molecule interactions with DNA and related biomacromolecules. For instance, several studies employed multi-spectroscopic DNA-binding analyses supported by computational approaches to clarify binding modes and interaction forces, highlighting best-practice frameworks for mechanistic interpretation^22,23^.

According to the available literature, this study represents the first systematic in vitro investigation of the binding interaction between DGV and SS-DNA. The novelty of the present work stems from combining complementary spectroscopic techniques (spectrophotometry and spectrofluorimetry) with ionic strength and viscosity analyses to comprehensively characterize the binding mechanism. In addition, molecular docking and thermodynamic evaluations were integrated to provide molecular-level insights into the binding site, affinity, and dominant interaction forces. This multifaceted approach submits a clear understanding of the DGV-DNA interaction, offering a reference platform for future pharmacological, clinical, and rational drug design studies targeting enhanced selectivity and therapeutic performance.

## Experimental

### Instrumentation

The spectrofluorimetric investigations were carried out on a Cary Eclipse fluorescence spectrophotometer (Agilent Technologies, USA), supplied with a xenon flash lamp adjusted at high sensitivity. The spectrophotometric studies were implemented using the Shimadzu ultraviolet-visible (UV-VIS) 1601 recording spectrophotometer (P/N 206 67001). Viscosity studies were performed using an Ostwald viscometer with a capillary inner diameter of 0.57 mm. A Consort NV P-901 pH meter (Belgium) for pH adjustments.

### Materials and reagents

Analytical grade chemicals and reagents were employed throughout the study.

Dolutegravir (purity ≥ 99.89%, as certified) was obtained from Beijing Mesochem Technology Co., Ltd. (Beijing, China, batch number HF200318DR). Tris(hydroxymethyl)aminomethane (Tris) and organic solvents were obtained from Fisher Scientific (UK). Salmon sperm DNA (Sigma Aldrich, USA), Ethidium bromide (EB) (Loba Chemie, India), and Rhodamine B (RB) (Yunbang Pharm, China) were used in the study. Hydrochloric acid, sodium chloride, and potassium iodide were obtained from El-Nasr Pharmaceutical Chemicals Co. (Cairo, Egypt).

An aqueous 0.05 M Tris–HCl buffer was employed, with the pH adjusted and maintained at 7.4 throughout the study using 1.0 M HCl. Stock solutions of RB and EB, each at 1.0 × 10^− 3^ M, were prepared in ethanol, whereas stock solutions of NaCl and KI, each at 1.0 M, were prepared in distilled water. All solutions were freshly prepared and stored at 4 °C.

### Standard solutions

A mass of 0.01 g of lyophilized SS-DNA was dissolved in 50 mL of Tris-HCl buffer (pH 7.4) to obtain the stock solution, which was kept under dark conditions for up to five days, with regular stirring to ensure homogeneity. Its purity was confirmed by the UV absorbance ratio (A_260_/A_280_) of 1.91 (above 1.8), indicating the absence of protein contamination. The concentration of the SS-DNA stock solution was computed from the absorbance at 260 nm (T = 298 K, ε_260_ = 6600 M⁻¹ cm⁻¹)^24–26^. The standard stock solution of DGV was prepared at a concentration of 250 µM using methanol in a 100 mL volumetric flask. All solutions were stored at 4 °C.

### Experimental procedures

#### Spectrophotometric studies

The thermodynamic study and computation of the binding constant were implemented through scanning the UV spectra of 45.4 µM SS-DNA solutions in the 200–400 nm range upon titrations with successive concentrations of DGV in the range of 0–25.0 µM at four distinct temperatures (302, 307, 314, and 320 K). The corrected UV–Vis spectrum was obtained by subtracting the absorbance spectrum of DGV alone from that of the DGV-DNA complex.

#### Ionic strength studies

The UV absorption spectra of the DGV-DNA complex were monitored at NaCl concentrations ranging from 0 to 0.07 M, while maintaining fixed concentrations of SS-DNA (45.4 µM) and DGV (20.0 µM).

#### Competitive displacement studies

Competitive displacement studies were conducted employing different DNA-interacting dyes, including EB and RB, in 0.05 M Tris-HCl buffer at pH 7.4. In EB experiments, a constant EB concentration of 10.0 µM was maintained, and its spectrofluorimetric emission was recorded before and after the addition of 1.4 µM DNA, at λ_ex/em_ of 525/603 nm. The fluorescence intensity of the DNA-EB complex was then measured after the addition of increasing concentrations of DGV (1.25–4.0 µM). For RB experiments, a fixed concentration of 1.0 µM RB was used, and its emission spectrum was measured before and after the addition of 1.4 µM DNA, at λ_ex/em_ of 465/576 nm. The fluorescence intensity of the DNA-RB complex was further monitored upon titration with successive DGV concentrations (1.25–4.0 µM).

#### Iodide quenching studies

Two experiments were conducted using a fixed concentration of DGV (3.75 µM) in 0.05 M Tris-HCl buffer at pH 7.4. In the first experiment, the fluorescence intensity of DGV was monitored upon incremental addition of KI at concentrations ranging from 0 to 40 × 10^− 3^ M, using the optimal excitation and emission wavelengths of DGV (262/415 nm). In the second experiment, the fluorescence intensity of DGV at the same fixed concentration was recorded in the presence of a constant SS-DNA concentration (0.37 µM) with incremental additions of KI. The quenching constants for both experiments were computed using the Stern-Volmer equation.

#### Viscosity measurements

At a constant temperature of 298 K, the Ostwald viscometer was used to perform the viscosity study. The study involved recording the flow times of solutions containing SS-DNA (45.4 µM) in 0.05 M Tris-HCl buffer at pH 7.4, in the presence of elevated concentrations of DGV, ranging from 0 to 25.0 µM. The flow times were monitored using a digital stopwatch in triplicate. Viscosity values (η) were determined following the formula: $\:{\upeta\:}=(\mathrm{t}-\mathrm{t}_{\circ})/\mathrm{t}_{\circ},$ where t and t₀ are the flow times of the SS-DNA and the buffer solutions, respectively^11,12^. The relative specific viscosity was expressed as (η/η_₀_)^1/3^, where η and η₀ denote the specific viscosities of SS-DNA in the presence and absence of DGV, respectively.

#### Docking studies

Three-dimensional structures of three B-DNA sequences—1BNA^27^, 1D29^28^, and 3EY0^29^ —were retrieved from the Protein Data Bank (PDB). Both the DNA targets and the ligand DGV were prepared using UCSF Chimera^30^prior to the docking process. The downloaded DNA helices were inspected to remove crystallographic water molecules and any non-DNA entities. Missing hydrogen atoms were added, and appropriate protonation states were assigned using UCSF Chimera. Partial atomic charges were applied using the AMBER ff14SB force field for nucleic acids. The prepared DNA structures were subsequently energy-minimized to relieve steric clashes while preserving the native B-DNA conformation before being used as rigid receptors in the docking simulations. The structure of DGV was obtained from the PubChem database and energy-minimized and prepared in Chimera prior to docking simulations. Preparation included assignment of partial atomic charges using the Gasteiger method for the ligand.

Molecular docking investigations were carried out using AutoDock Vina^31,32^to elucidate the binding modes of DGV to three B-DNA sequences. Docking was performed using AutoDock Vina’s Monte Carlo iterated local search global optimization algorithm with exhaustiveness of search = 8, number of binding modes = 10, and energy range = 3 kcal/mol. Table 1 demonstrates the grid box used for each DNA helix. Top-ranked poses were selected based on binding energy (ΔG in kcal/mol) and visual inspection of hydrogen bonding and minor groove localization. Detailed interaction analysis was performed using Chimera and the Protein-Ligand Interaction Profiler^33^(PLIP) web tool. Two-dimensional interaction diagrams were generated using ProteinsPlus^34–36^to visualize the key interactions between the ligand and DNA bases in the binding sites.

**Table 1:** PDB entries and the grid box specifications for the three DNA sequences employed for the molecular docking analysis.

DNA (PDB ID)	Sequence	Grid box center coordinates (x, y, z)	Grid box size (Å) (x, y, z)
**1BNA**	5′-(CGC GAA TTC GCG)−3′	16.3, 24.2, 9.19	20, 20, 40
**1D29**	5′-(CGT GAA TTC ACG)−3′	16.7, 22.3, 8	18, 23, 41
**3EY0**	5′-(ATA TAT ATAT)−3′	15.8, 10.7, 90.7	33, 22, 33

## Results and discussion

### UV-Visible study

**Fig. 1:**
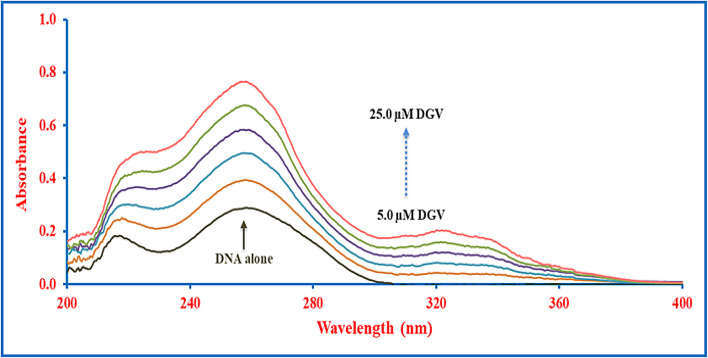
UV absorption spectra of SS-DNA (45.4 µM) titrated with increasing concentrations of DGV (0–25.0 µM).

UV-Visible spectroscopy is a straightforward and informative assay for inferring both qualitative and quantitative details about how small molecules bind to DNA. Such interactions occur through three main non-covalent mechanisms: intercalation, groove binding, and electrostatic interactions^37^. Upon the incremental addition of the binder to DNA, intercalators produce a hypochromic effect, along with a noticeable bathochromic shift. This comes into account due to coupling between the DNA’s π-antibonding orbital and the drug’s π-bonding orbital, which decreases the π–π* transition energy and causes the absorption band to shift toward longer wavelengths. Conversely, groove binders interact primarily within the grooves of DNA, where the electronic environment of the molecule’s chromophore overlaps weakly with that of the nitrogenous bases, often resulting in a mild hyperchromic effect with little to no shift in the absorption maximum^6,21,38,39^. As represented in Fig. 1, the incremental addition of DGV to DNA results in a hyperchromic effect with a constant band position, indicating that DGV binds to DNA *via* groove-mode or electrostatic interactions. Moreover, the corrected (difference) UV spectrum of the drug–DNA system was calculated to eliminate the intrinsic absorbance contribution of DGV, thereby allowing accurate evaluation of its effect on DNA. The corrected spectrum exhibited only a slight change in absorbance relative to native DNA, with minimal or no shift in wavelength **(Fig. S2)**. Such spectral behavior is consistent with a groove-binding mode and is inconsistent with intercalative binding, which is typically associated with large absorbance variations and pronounced bathochromic shifts^40^.

To better clarify the interaction mode and binding constant of DGV with DNA, spectrophotometric monitoring of the absorbance of SS-DNA (45.4 µM) upon titration with successive concentrations of DGV (0–25.0 µM) was performed at four distinct temperatures (302, 307, 314, and 320 K)^15^. The experiments included recording the absorption spectra in Tris-HCl buffer at pH 7.4 and a constant concentration of DNA (45.4 µM). Relying on the modified Benesi–Hildebrand equation, the interaction data were calculated as follows^6,39^:

1 $$\:\frac{\mathrm{A}^\circ\:}{\mathrm{A}-\mathrm{A}^\circ\:}=\frac{{\varepsilon\:}\mathrm{D}\mathrm{N}\mathrm{A}}{{\varepsilon\:}\mathrm{D}\mathrm{G}\mathrm{V}\_\mathrm{D}\mathrm{N}\mathrm{A}-{\varepsilon\:}\mathrm{D}\mathrm{N}\mathrm{A}}+\frac{{\varepsilon\:}\mathrm{D}\mathrm{N}\mathrm{A}}{{\varepsilon\:}\mathrm{D}\mathrm{G}\mathrm{V}\_\mathrm{D}\mathrm{N}\mathrm{A}-{\varepsilon\:}\mathrm{D}\mathrm{N}\mathrm{A}}\times\:\frac{1}{\mathrm{K}\mathrm{b}\times\:\left[\mathrm{D}\mathrm{G}\mathrm{V}\right]}$$

**Fig. 2:**
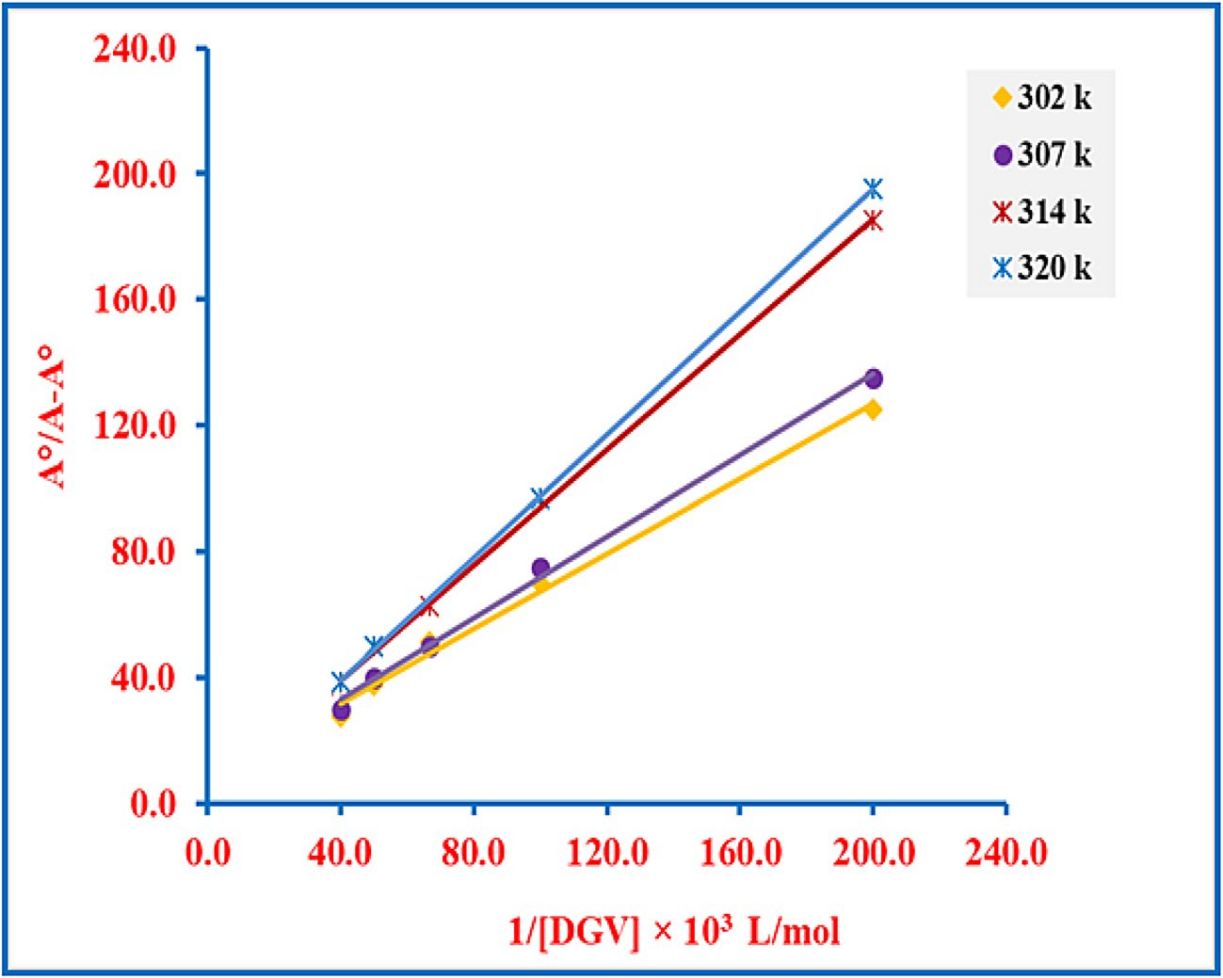
Plot of A°/(A – A°) versus 1/[DGV] at different temperatures, using SS-DNA (45.4 µM) and DGV concentrations ranging from 0 to 25.0 µM.

Where A$\vphantom{0}^\circ\:$ represents the absorption of SS-DNA (45.4 µM) at 260 nm, $\:\mathrm{A}\:$denotes the absorption of the same concentration of SS-DNA in the presence of increasing concentrations of DGV, and $\:{\mathrm{K}}_{\mathrm{b}}\:$is the binding constant for the DGV-DNA interaction. $\:{\varepsilon\:}_{\mathrm{DNA}}$and $\:{\varepsilon\:}_{\mathrm{DGV-DNA}}$correspond to the molar absorption coefficients of free DNA and the DGV-DNA complex, respectively. For all studied temperatures, plotting A^o^/(A–A^o^) *versus* 1/[DGV] reveals a good linear relationship (> 0.99), suggesting that the DGV-DNA complex follows a 1:1 binding stoichiometry (Fig. 2).

Additionally, K_b_ values were calculated at each temperature from the intercept-to-slope ratio of Eq. 1. According to the data listed in Table 2, the calculated K_b_ values were in the order of 10^3^ M^− 1^, indicating a modest binding of DGV to DNA. Although these values are lower than those reported for classic minor groove binders, including Hoechst derivatives and Netropsin, which typically exhibit binding constants in the range of 10⁷–10⁸ M^− 1^ through a combination of hydrogen bonding and van der Waals forces^41–43^, they are still consistent with a groove-binding mode^44–46^. Collectively, these findings evidenced that DGV binds to DNA through groove binding.

**Table 2:** Binding constants at four different temperatures and thermodynamic parameters of the DNA-DGV complex.

Temperature K	K_b_×10^3^ M^−1^	*r*	∆H° kJ mol^−1^	∆S° J mol^−1^ K^−1^	∆G° kJ mol^−1^
302	13.695	0.9966	−198.51	−573.33	−25.4
307	10.669	0.9984			−22.5
314	2.499	0.9997			−18.5
320	0.142	0.9999			−15.0

### Ionic strength assay

Salt concentration-dependent experiments have served as a crucial tool in investigating the likelihood of electrostatic interaction between a drug and DNA. In this study, the absorbance spectra of the DGV-DNA complex were obtained after titration with varying concentrations of strong electrolyte (e.g., NaCl) to assess the effect of ionic strength. As reported in the literature^16,47^, elevated concentrations of cations (Na^+^) interact with the negatively charged phosphate backbone of SS-DNA, thereby weakening the electrostatic binding at the DNA surface. This effect manifests as a reduction in spectral intensity for the targeted molecules bound to SS-DNA through electrostatic interactions. As demonstrated in Figs. S3 and S4, the recorded absorbance of the complex exhibited a consistent trend at elevated NaCl levels, which rules out the contribution of electrostatic interactions in the binding of DGV to SS-DNA.

### Competitive displacement assay

**Fig. 3:**
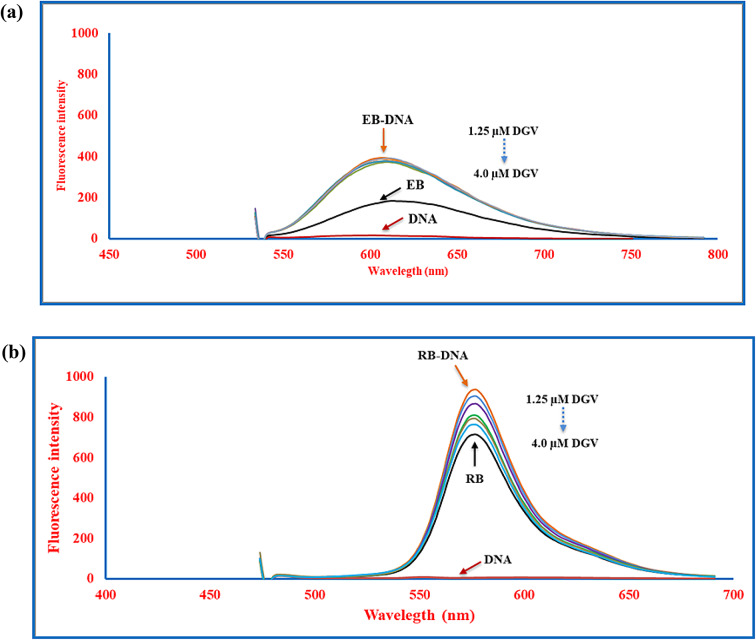
**(a)**: Fluorescence emission spectra of the EB-DNA complex recorded at 298 K in both the absence and presence of increasing concentrations of DGV. Experimental conditions were as follows: SS-DNA, 1.4 µM; EB, 10.0 µM; DGV, 1.25–4.0 µM, with excitation and emission wavelengths set at λ_ex/em_ 525/603 nm. (**b)**: Fluorescence emission spectra of RB-DNA complex recorded at 298 K in both the absence and presence of increasing concentrations of DGV. Experimental conditions were as follows: SS-DNA, 1.4 µM; RB, 1.0 µM; DGV, 1.25–4.0 µM, with excitation and emission wavelengths set at λ_ex/em_ 465/576 nm.

Fluorescence spectroscopy is a premier approach for exploring the interaction mode between DNA and small molecules. In this study, two reference dyes with distinct DNA binding modes are used following competitive displacement experiments. EB is a weakly fluorescent dye that exhibits a significant increase in fluorescence when it interacts with DNA base pairs through intercalation, using λ_ex/em_ of 525/603 nm. The addition of an intercalating agent typically displaces EB from DNA, leading to a pronounced decrease in fluorescence intensity^6,21^. In the suggested study, upon titrating the EB-DNA solution with increasing concentrations of DGV (1.25**–**4.0 µM), nearly constant measurements were observed, indicating that DGV is not likely to bind to DNA through intercalation (Fig. 3a).

In contrast to RB, which is known in the literature for its groove binding with DNA base pairs. RB is a sensitive fluorescent probe whose fluorescence intensity increases upon interaction with DNA, using λ_ex/em_ of 465/576 nm. It is well established that groove binder molecules compete with RB in its complex with DNA, causing a decline in fluorescence intensity^48,49^. As depicted in Fig. 3b, incremental addition of DGV (1.25**–**4.0 µM) to RB-DNA solutions causes a substantial reduction in the fluorescence intensity of the RB-DNA complex, indicating the competitive replacement of RB by DGV in its DNA binding.

These outcomes collectively support the interaction of DGV with DNA *via* a groove-binding mode.

### Iodide quenching studies

Potassium iodide (KI) quenching was employed as a well-established spectrofluorimetric tool to probe the accessibility of DGV in both its free and DNA-bound states^13^. Fluorescence measurements of DGV, both in free and bound states, were monitored during titration with increasing KI concentrations at λ_ex/em_ of 262/415 nm. The Stern-Volmer formula was then executed to compute the quenching constant in both states as follows:

2 $$\:\mathrm{F}^{\circ}/\mathrm{F}\:=\:1+\:\mathrm{K}\mathrm{s}\mathrm{v}\:\left[\mathrm{K}\mathrm{I}\right]\:$$

Where F° is the fluorescence signal without the addition of KI, and F denotes the fluorescence after adding KI, [KI] is the molar concentration, and K_sv_ is the quenching constant. Typically, groove binders are equally accessible to iodide quenchers, in both free and DNA-bound states, as their surface location on the DNA allows unhindered attack from negatively charged quenchers, despite DNA binding, thus affecting their fluorescence intensity.

**Fig. 4:**
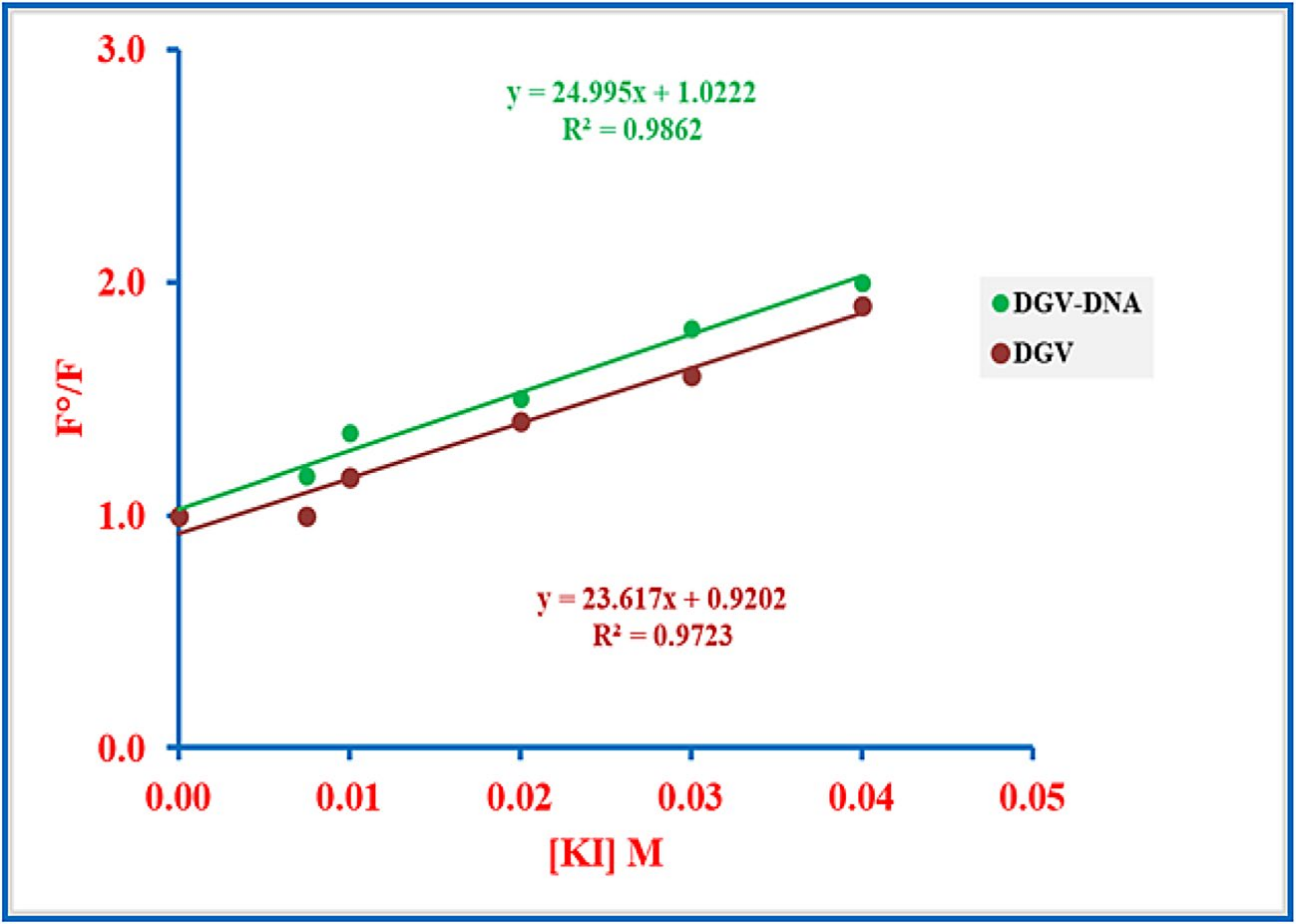
Stern-Volmer plot for fluorescence quenching of DGV (3.75 µM) by increasing concentrations of KI in the absence and presence of SS-DNA (1.4 µM).

Conversely, intercalators are protected within DNA base pairs and shielded by the electrostatic repulsion of iodide ions with the negatively charged DNA backbone, leading to minimal fluorescence quenching^50,51^. The results, shown in Fig. 4, indicate K_sv_ values of 24.99 and 23.61 M^− 1^ in the presence and absence of DNA, respectively. The small difference between these values strongly supports the groove-binding mode, thereby excluding intercalation as the binding mechanism.

### Viscosity measurements

For a more profound investigation of the binding mechanism of DGV with SS-DNA, viscosity studies were performed. Viscosity, a pivotal hydrodynamic parameter, provides a straightforward yet informative approach to probing the binding modes of small molecules to DNA^11,12,52^. Typically, classical intercalators extend the DNA helix by inserting themselves between base pairs, which separates the pairs at the intercalation sites and leads to an increase in the DNA’s relative specific viscosity. In contrast, groove binders generally maintain the relative specific viscosity unchanged, as they associate through the grooves without significantly affecting the helix length, resulting mainly in bending or twisting of the helix. As illustrated in the viscosity plot (**Fig. S5**), increasing concentrations of DGV induce a negligible change in the DNA’s relative viscosity, a hallmark of the minor groove binding mode^53,54^.

### Thermodynamic parameters

DNA interacts with ligands primarily through non-covalent forces, comprising hydrogen bonding, van der Waals forces, and hydrophobic and electrostatic interactions. Thermodynamic quantities such as enthalpy (ΔH^o^), entropy (ΔS^o^), and Gibbs free energy (∆G°) serve as molecular fingerprints, revealing both the driving forces and the spontaneity of complex formation^55^. To unravel the interaction energetics of the DGV-DNA complex, the Van’t Hoff formula was executed^12^:

3 $$\:\mathrm{l}\mathrm{n}\:\mathrm{K}\mathrm{b}\:=\:-\:({\Delta\:}\mathrm{H}^\circ\:\:/\mathrm{R}\mathrm{T})\:+\:({\Delta\:}\mathrm{S}^\circ\:\:/\mathrm{R})$$

 

Where K_b_ is the binding constant, R is the universal gas constant (8.314 J K^− 1^ mol^− 1^), and T is the absolute temperature in kelvin.

**Fig. 5:**
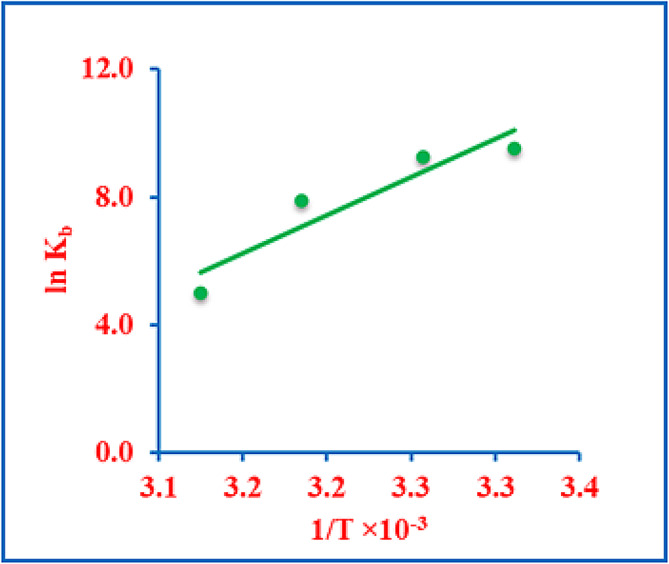
Van’t Hoff plot for the DNA-DGV complex.

DNA absorbance was monitored at four temperatures (302, 307, 314, and 320 K) with increasing DGV concentrations. Plotting ln K_b_ against 1/T allowed determination of ∆H° (from the slope) and ∆S° (from the intercept) (Fig. 5). To find ∆G°, the following equation was used^6^:

4 $$\:\varDelta\:\mathrm{G}^\circ\:=\:\varDelta\:\mathrm{H}^\circ\:-\:\mathrm{T}\varDelta\:\mathrm{S}^\circ\:$$

As demonstrated in Table 2, all thermodynamic parameters (∆H°, ∆S°, and ∆G°) exhibit negative values. These signs provide insight into the interaction forces; negative ∆H° (−198.51 kJ mol^− 1^) and ∆S° (−573.33 J mol^− 1^ K^− 1^) are indicative of van der Waals interactions, and hydrogen bonds as the dominant stabilizing forces in the DGV-DNA complex^15,56^. The negative ∆G° (−15.0 to −25.4 kJ mol^− 1^) indicates that the binding process is spontaneous and thermodynamically favorable, consistent with a well-ordered binding mode within the DNA architecture.

### Molecular docking

Molecular docking serves as a crucial tool in drug discovery, commonly used to understand and forecast ligand-receptor interactions. It offers valuable insights that help validate experimental results and direct rational drug design. In this context, docking studies were conducted to explore how DGV interacts with DNA using three B-DNA sequences (1BNA, 1D29, 3EY0). These sequences are well-established B-form DNA duplexes, often used as representative models to study binding sites and modes of small-molecule probes within SS-DNA^6,39,56^. Specifically, 1BNA is a canonical B-DNA dodecamer presenting both major and minor grooves. Due to its structural stability and high-resolution crystallographic data, 1BNA has been employed as a reference model for evaluating the DNA-binding potential of small molecules, especially those that may intercalate or bind within the grooves^57^. PDB IDs 1D29 and 3EY0 were selected due to their high-resolution crystallographic data and reliable DNA conformations. These structures provide additional B-DNA duplex models with different base sequences and groove geometries, allowing assessment of DGV binding consistency across diverse DNA environments^6,39,56^. The selection of multiple DNA models ensures a reliable, reproducible, and comprehensive evaluation and minimizing sequence- or structure-dependent biases.

Computational modelling outcomes revealed that DGV predominantly binds within the minor groove of B-DNA, especially in GC-rich regions, in alignment with findings from fluorescence probe studies and UV-visible spectrophotometric analyses.

**Fig. 6:**
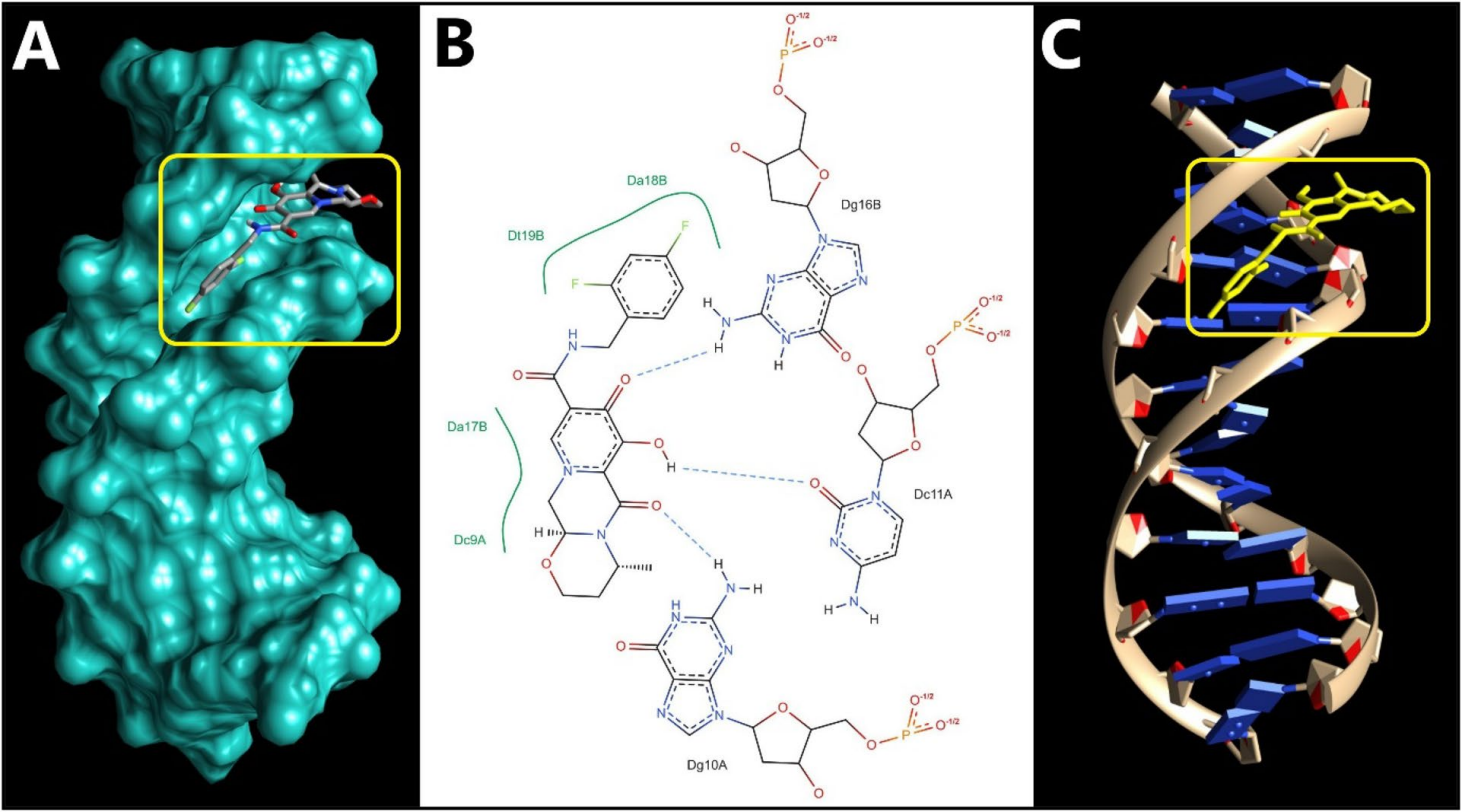
Predicted binding mode of DGV with the DNA duplex (PDB: 1BNA). **(A)** Surface representation of the DNA helix (in cyan) showing DGV (sticks model) bound within the minor groove of the DNA; the yellow box highlights the binding pocket. **(B)** 2D diagram of interactions between DGV and DNA bases, including hydrogen bonds (dashed blue lines) and hydrophobic contacts (green arcs). **(C)** Cartoon representation showing DGV highlighted in a yellow stick model within the DNA helix.

As shown in Fig. 6, binding of DGV occurs in the minor groove of B-DNA duplex (PDB: 1BNA) with a binding energy of −8.08 kcal/mol, forming three hydrogen bonds with both strands’ nucleotides. The most favorable interaction was with DG10A, in which it formed a good hydrogen bond with the bond length 2.00 Å and bond angle 157.7°, which is likely to be the prime anchor in the groove. Additionally, two other hydrogen bonds were found with DC11A at a distance of 2.67 Å and an angle of 123.6° and with DG16B at a distance of 2.33 Å and with an angle of approximately 109°, representing more surface stabilization-related interactions than deep insertion, enhancing the ligand’s alignment within the groove. In addition to these polar contacts, the fluorinated aromatic ring and the adjacent fused ring system of DGV exhibited hydrophobic and π–π stacking interactions with neighboring bases DC9A, DA17B, DA18B, and DT19B, thereby anchoring its groove-bound conformation. These contacts demonstrate an unambiguous minor groove binding mode, combining both hydrogen bonding and hydrophobic contact to achieve stable DNA association.

**Fig. 7:**
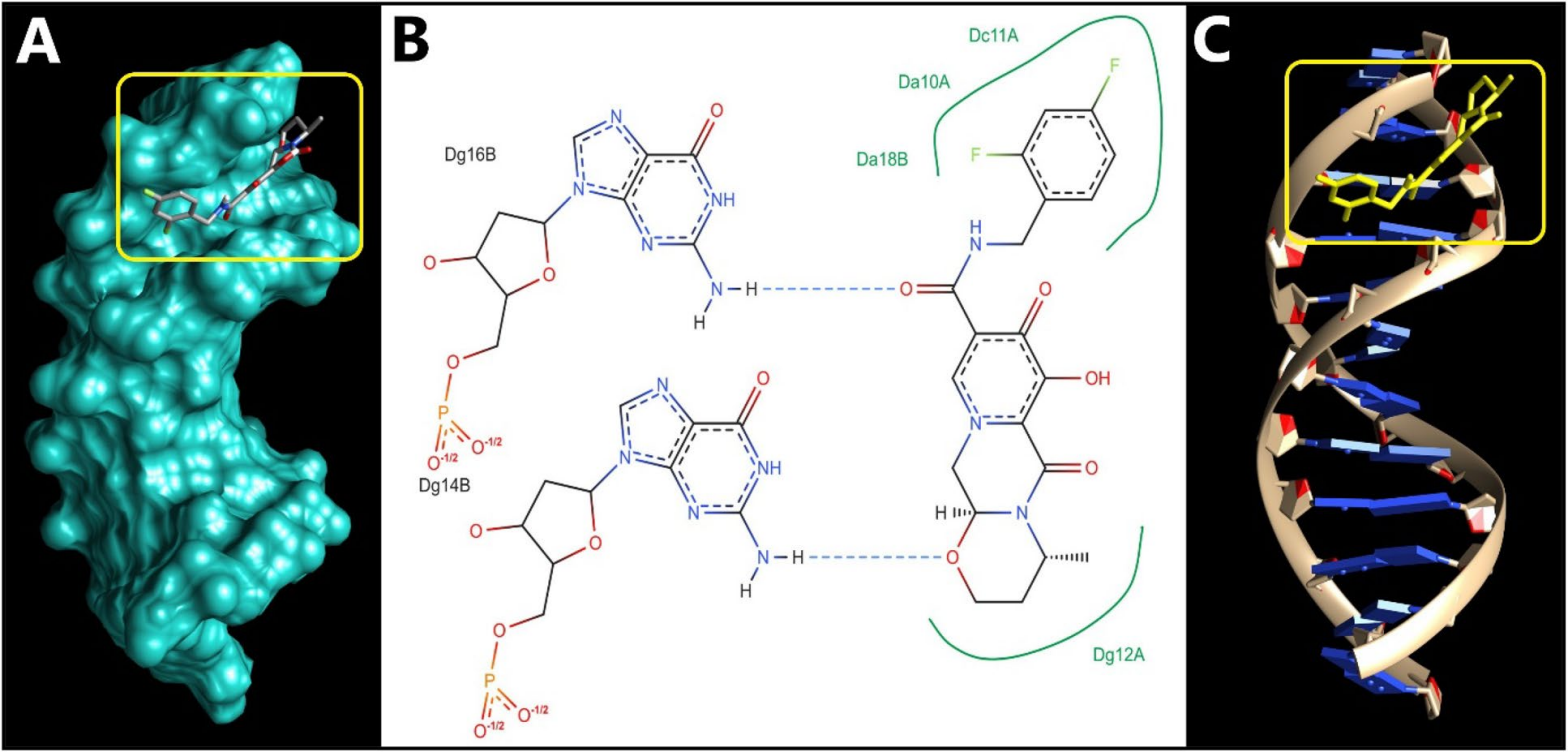
Predicted binding mode DGV with the DNA duplex (PDB ID: 1D29). **(A)** Surface representation of the DNA helix (in cyan) showing DGV (sticks model) bound within the minor groove of the DNA; the yellow box highlights the binding pocket. **(B)** 2D diagram of hydrogen bonds (dashed blue lines) and hydrophobic contacts (green arcs) between DGV and DNA bases. **(C)** Cartoon representation showing DGV highlighted in a yellow stick model within the DNA helix.

DGV docking onto the DNA duplex of PDB: 1D29 (Fig. 7) showed that it binds mostly with guanine-rich regions with a binding energy equals − 7.25 kcal/mol, where two well-defined hydrogen bonds were present. The initial one was with guanine at position 14B, bond length 2.08 Å, and bond angle 158.8°, which makes it a highly directional bond, while the second one was with guanine at position 16B, bond length 2.09 Å, and angle 156.7°. In addition to these hydrogen bonds, the interaction map suggests potential π–π stacking and hydrophobic contacts between the aromatic fluorinated ring of the ligand with several purine bases, particularly DC11A, DA10A, DA18B, and DG12A.

**Fig. 8:**
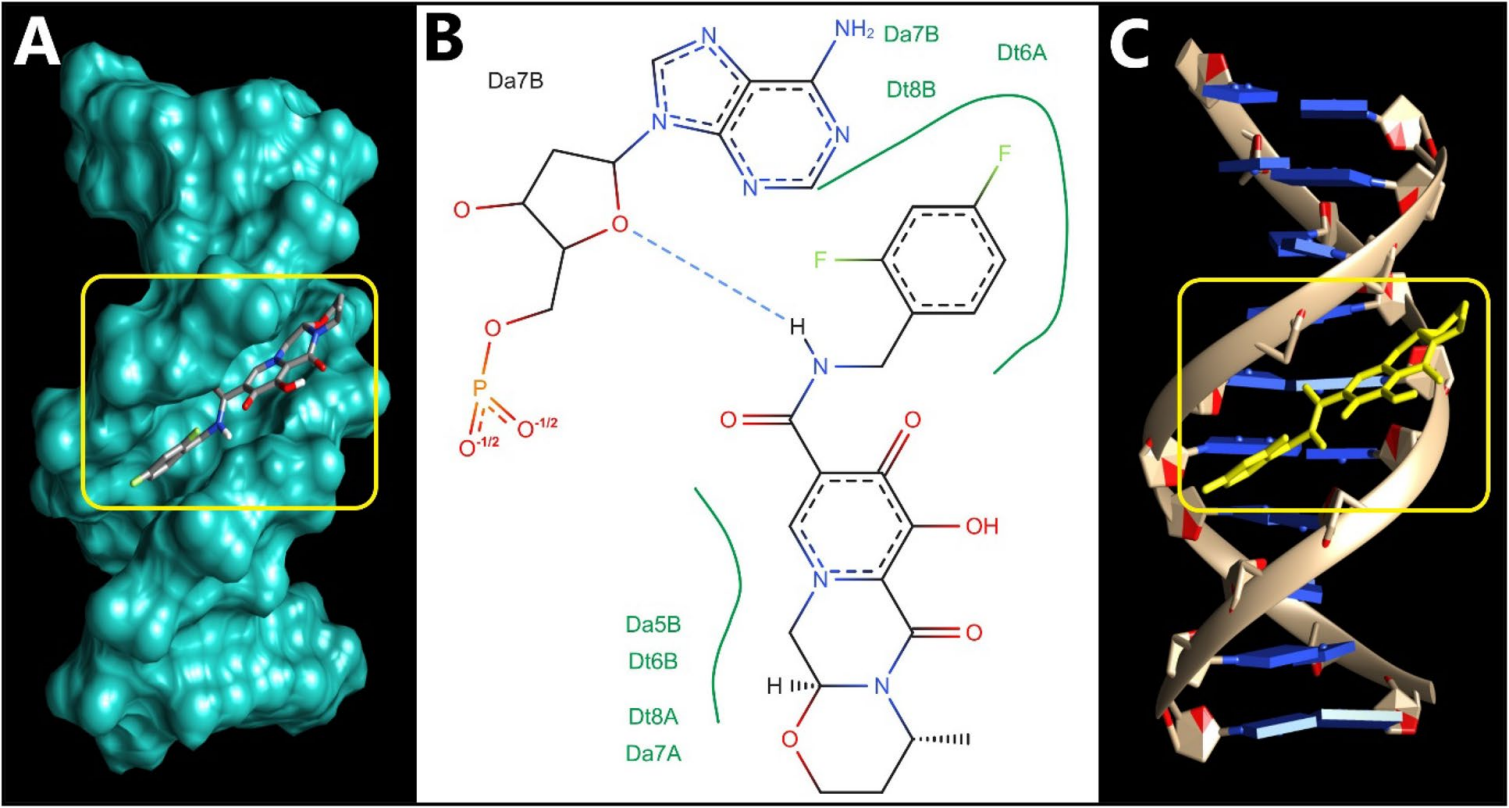
Predicted binding mode DGV with the DNA duplex (PDB ID: 3EY0). **(A)** Surface representation of the DNA helix (in cyan) showing DGV (sticks model) bound within the minor groove of the DNA; the yellow box highlights the binding pocket. **(B)** 2D diagram detailing hydrogen bond (dashed blue line) and multiple hydrophobic contacts (green arcs) between the ligand and DNA. **(C)** Cartoon representation showing DGV highlighted in a yellow stick model within the DNA helix.

Best docking pose of DGV with the DNA duplex of PDB: 3EY0 (Fig. 8) exhibited a single but strong hydrogen bond with adenine at position 7B with a bond length of 2.17 Å and a favorable angle of 140°, suggesting a stable and directional hydrogen bond. Beyond this polar interaction, the binding appears to be further stabilized by extensive hydrophobic contacts. The fluorinated phenyl ring of DGV is nestled among several base residues, including thymine DT6A, DA7B, and DT8B, forming a compact hydrophobic patch. On the opposite end of the molecule, additional contacts were observed with DA5B, DT6B, DA7A, and DT8A, contributing to a broader base stacking environment; the binding energy was − 8.57 kcal/mol.

Collectively, the binding orientations observed for the three DNA complexes indicate that DGV preferentially binds within the minor groove of the DNA duplex rather than intercalating between base pairs. The interactions are a combination of directional hydrogen bonds and extensive hydrophobic contacts that span both strands to further stabilize a groove-aligned conformation. Such a binding mode is also supported by the geometry of DGV, which allows it to closely adapt over the groove surface without sacrificing the base stacking or helical integrity of DNA.

To facilitate comparison across the three DNA models, a summary of docking energies and key polar interactions is provided (Table 3). Across all systems, DGV consistently adopts a minor-groove binding pose, supporting the experimentally inferred groove-binding mode. Notably, for the GC-containing helices (1BNA and 1D29), the most stabilizing polar contacts involve guanine/cytosine positions (DG and DC bases), with short hydrogen-bond distances (2.00–2.67 Å), suggesting that GC-associated edges can provide favorable hydrogen-bonding opportunities for DGV. This offers a structural rationale for the observed preference toward GC-rich regions reported in our docking analysis. In contrast, the AT-rich duplex (3EY0) still supports a stable minor-groove pose but is stabilized largely through extensive hydrophobic packing and a single hydrogen bond, indicating that DGV can bind groove sites with different sequence composition while displaying stronger polar anchoring in GC-containing sites. Overall, these sequence-dependent interaction patterns are consistent with groove-binding literature, where ligand shape complementarity within the groove and the availability of suitable hydrogen-bond donors/acceptors govern sequence selectivity and binding stabilization.

**Table 3:** Summary of docking results for DGV with three B-DNA sequences.

DNA (PDB ID)	Binding energy (kcal/mol)	Hydrogen-bond partners (DNA base)	H-bond distance(s) (Å)	Key additional hydrophobic/π contacts
**1BNA**	−8.08	DG10A, DC11A, DG16B	2.00, 2.67, 2.33	DC9A, DA17B, DA18B, DT19B
**1D29**	−7.25	DG14B, DG16B	2.08, 2.09	DC11A, DA10A, DA18B, DG12A
**3EY0**	−8.57	DA7B	2.17	DT6A, DA7B, DT8B, DA5B, DT6B, DA7A, DT8A

The molecular docking results provide a strong structural basis for experimental DNA-binding observations. Spectroscopic studies demonstrated that DGV interacts with SS-DNA via a non-intercalative groove-binding mode. Consistently, docking simulations reveal that DGV preferentially localizes within the minor groove of B-DNA, forming hydrogen bonds and hydrophobic contacts with nucleobases, without insertion between base pairs or perturbation of the DNA helical structure. Moreover, the dominance of hydrogen bonding and van der Waals interactions observed in docking correlate well with the negative ΔH° and ΔS° values obtained from thermodynamic analysis, collectively confirming minor groove binding as the primary interaction mode.

This integrated experimental–computational interpretation is consistent with recent DNA-binding studies that combine spectroscopic evidence with docking/simulation tools to support binding-site preference and dominant stabilizing forces.

### Comparison with previous studies and mechanistic insights

The interaction between DGV and DNA was systematically investigated using a combination of complementary techniques to elucidate the binding mechanism. Collectively, the obtained results consistently support a groove-binding mode rather than classical intercalation.

UV absorption studies revealed that the interaction of DGV with DNA induced little to no shift in the absorption maxima, suggesting groove or external electrostatic interactions. This observation was further supported by the minor changes observed in the difference absorption spectra, in contrast to the pronounced absorbance variations typically associated with classical intercalators. The binding constant was estimated to be on the order of 10^3^ M⁻¹, which, although slightly lower than that reported for some groove binders^41–43^, remains within the expected range for groove-mediated interactions^44–46^. Moreover, the minimal effect of increasing ionic strength during titration indicates that electrostatic forces are not the primary driving factor governing the interaction.

Fluorescence-based experiments provided additional insight into the binding characteristics. Competitive displacement assays demonstrated that DGV effectively displaced RB from the RB-DNA complex, as evidenced by a marked decrease in fluorescence intensity, while no significant fluorescence reduction was observed for the EB-DNA complex^6,21,39^. Furthermore, Stern–Volmer quenching studies using KI suggested that DGV is not shielded between DNA base pairs, evidenced by nearly identical quenching constants. Consistent with these findings, viscosity measurements showed no significant change in DNA viscosity upon increasing DGV concentration, supporting a groove-binding mode that preserves the native DNA length^53,54^. These experimental observations were further corroborated by molecular docking studies, which confirmed the preferential insertion of DGV into the minor groove of the DNA duplex.

When contextualized within the existing literature, the DNA-binding behavior of DGV aligns well with previously reported non-intercalative small-molecule DNA binders^6,21,39^. Collectively, the spectroscopic, competitive binding, and computational results provide a cohesive and mechanistically consistent description of the DGV–DNA interaction.

These genomic interaction findings suggest that DGV may induce only subtle conformational changes in DNA, which may slightly modulate DNA–protein interactions without causing significant genomic instability. This is supported by the observed reduction in RAG1 binding to DNA substrates involved in V(D)J recombination, indicating limited effects on gene expression during long-term therapy. Importantly, these DNA-binding properties can be leveraged in oncology, as DGV has been shown to inhibit HERV-K expression and target proteins such as CKS2 in cancer cells, thereby reducing proliferation, migration, and invasion. Collectively, these results highlight a dual mechanism for DGV, combining minor-groove DNA interaction and modulation of protein targets, supporting its potential for repurposing as an anticancer agent.

### Limitations and future perspectives

The present study employed SS-DNA as an affordable and abundant alternative to mammalian genomic DNA model to evaluate the binding of DGV with DNA. While SS-DNA shares similar GC content with some mammalian genomes, oligonucleotide sequence features and base compositions are only partially related to GC content, and do not capture the full complexity of human genomic sequences. These findings highlight the need for future follow-up studies, including cellular assays such as the comet assay and gene expression profiling, to assess the potential genotoxicity and functional impact of DGV-DNA interactions. Such investigations would help bridge the gap between the current in vitro results and physiological relevance.

## Conclusion

The presented framework comprehensively explores how DGV interacts with SS-DNA, using a combination of spectroscopic experiments and molecular modelling. Ionic strength experiments indicated that electrostatic interactions are unlikely to be involved in the binding. Competitive displacement tests with RB and EB unveiled that DGV binds within the minor grooves of DNA. UV titration studies yielded a binding constant on the order of 10^3^ M⁻¹, corroborating the groove binding interaction. The KI quenching constants of DGV were determined to be 24.99 M⁻¹ and 23.61 M⁻¹ in the presence and absence of DNA, respectively. Viscosity measurements further supported this binding mechanism. Thermodynamic analysis revealed that the interaction is exothermic and spontaneous, as indicated by ∆G° values ranging from − 15.0 to −25.4 kJ mol⁻¹. The binding is predominantly stabilized by van der Waals forces and hydrogen bonds, as supported by the negative ∆H° (−198.51 kJ mol⁻¹) and ∆S° (−573.33 J mol⁻¹ K⁻¹) value. In silico molecular modelling provided additional confirmation of DGV’s minor groove binding to SS-DNA. Overall, this study clarifies the binding mechanism of DGV with SS-DNA and underscores the need for further studies on its toxicological and pharmacological implications.

## Supplementary Information

Below is the link to the electronic supplementary material.

Supplementary Material 1

## Data Availability

The datasets generated during the proposed study are available from the corresponding author on reasonable request. **Eman Yosrey** [eman_yosrey55@mans.edu.eg].
