# Supplementary material for: Deciphering the groove-binding mode of dolutegravir with salmon sperm DNA through spectroscopic and molecular modelling approaches
Source: Sci Rep. 2026 Mar 13;16:9092. doi: 10.1038/s41598-026-40136-y (PMC12992605; doi:10.1038/s41598-026-40136-y)
Supplement: Supplementary file 1 — Supplementary Material 1 [file 41598_2026_40136_MOESM1_ESM.docx]

**Deciphering the Groove-Binding Mode of Dolutegravir with Salmon Sperm DNA through Spectroscopic and Molecular Modelling Approaches**

**Eman Yosrey*^1^, Mohammad A. Elmorsy^2,3^, Heba Elmansi^1^,** [**Shereen**](https://analyticalsciencejournals.onlinelibrary.wiley.com/action/doSearch?ContribAuthorStored=Sheribah%2C+Zainab) **Shalan^1^, Jenny Jeehan Nasr^1^**

1. Department of Pharmaceutical Analytical Chemistry, Faculty of Pharmacy, Mansoura University, Mansoura 35516, Egypt
2. Department of Pharmaceutical Organic Chemistry, Faculty of Pharmacy, Mansoura University, Mansoura 35516, Egypt
3. Department of Pharmaceutical Chemistry, Faculty of Pharmacy, Delta University for Science and Technology, Gamasa City 11152, Dakahliya, Egypt

* Corresponding author:

E-mail address: [eman_yosrey55@mans.edu.eg](mailto:eman_yosrey55@mans.edu.eg) / [emanyosrey435@gmail.com](mailto:emanyosrey435@gmail.com)

**Figure S1:** Chemical structure of DGV.

**Figure S2:** UV-visible absorption spectra of DGV (20.0 µM), DNA (45.4 µM), and the corrected (difference) absorption spectrum of the DGV-DNA complex.

**Figure S3:** Effect of NaCl ionic strength on the full UV–visible absorption spectra of the DGV-DNA complex, recorded using DGV and SS-DNA concentrations of 20.0 µM and 45.4 µM, respectively**.**

**Figure S4:** Effect of NaCl ionic strength on the absorbance of the DGV-DNA complex at 260 nm, measured using DGV and SS-DNA concentrations of 20.0 µM and 45.4 µM, respectively.

**Figure S5:** Influence of variable concentrations of DGV (0–25.0 μM) on the viscosity of SS-DNA (45.4 µM) in Tris–HCl buffer.


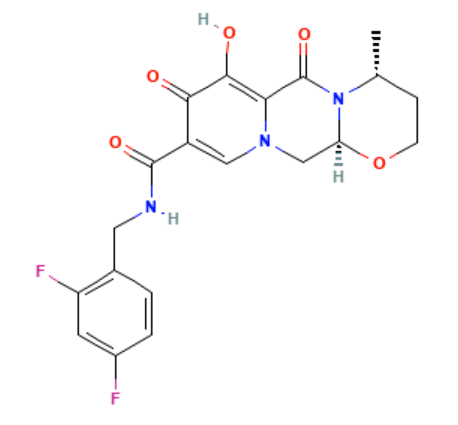


**Fig. S1**

**
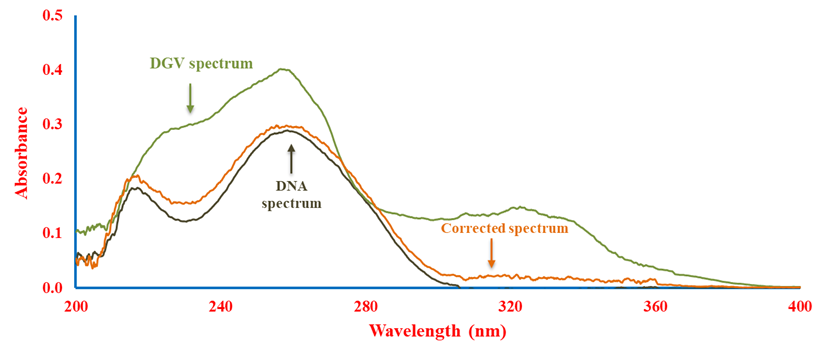
**

**Fig. S2**

**
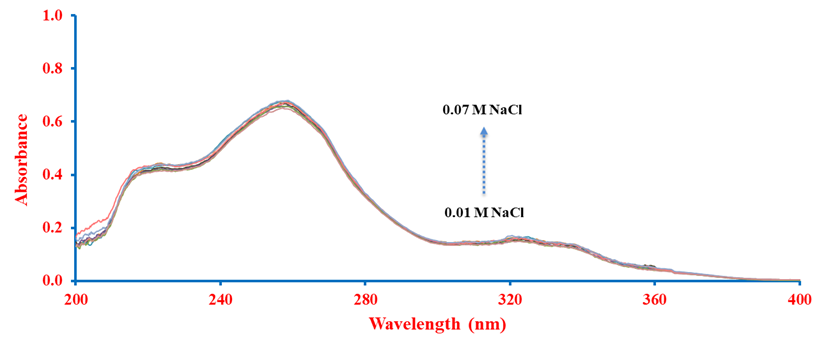
**

**Fig. S3**


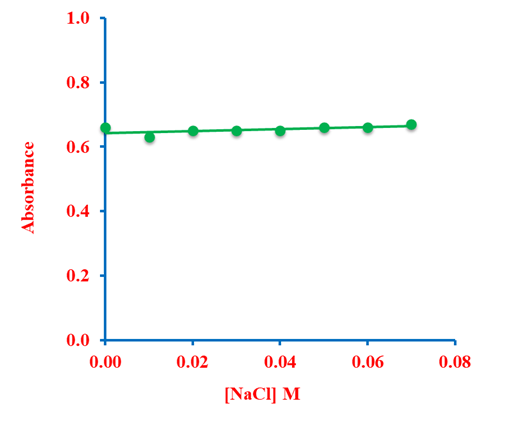


**Fig. S4**


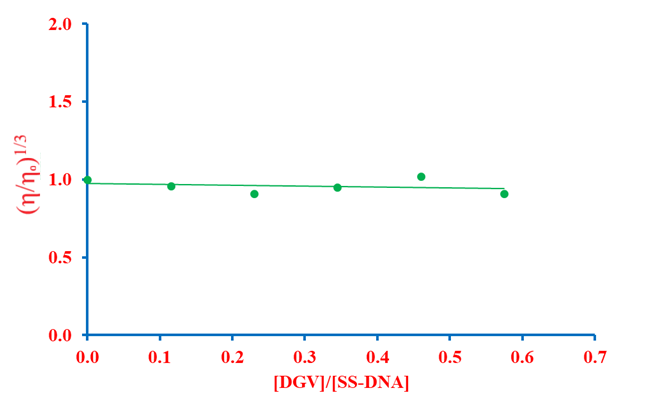


**Fig. S5**
